# Heritability and shared environmental effects of brain diseases in 12,040 extended families

**DOI:** 10.1038/s44400-025-00030-2

**Published:** 2025-11-03

**Authors:** Janna I. R. Dijkstra, Marc Hulsman, Lisa Waterink, Henne Holstege, Charlotte E. Teunissen, Wouter F. L. Christiaansen, Bryan A. de Jong, Peter Kochunov, Brian Donohue, Marissa D. Zwan, Anouk den Braber, Lisa Vermunt, Sven J. van der Lee

**Affiliations:** 1 Genomics of Neurodegenerative Diseases and Aging, Human Genetics, Vrije Universiteit, Amsterdam UMC location VUmc, Amsterdam, The Netherlands; 2 Alzheimer Center Amsterdam, Neurology, Vrije Universiteit, Amsterdam UMC location VUmc, Amsterdam, The Netherlands; 3 Neurochemistry Laboratory, Department of Laboratory Medicine, Amsterdam Neuroscience, Amsterdam UMC Vrije Universiteit, Amsterdam, The Netherlands; 4 Amsterdam Neuroscience, Neurodegeneration, Amsterdam, The Netherlands; 5 Psychiatric Research Center, University of Maryland Baltimore, Baltimore, MD USA; 6 Department of Psychiatry and Behavioral Science, University of Texas Health Science Center Houston, Houston, TX USA; 7 Department of Radiology and Nuclear Medicine, Vrije Universiteit, Amsterdam UMC location VUmc, Amsterdam, The Netherlands

**Keywords:** Dementia, Neurodegenerative diseases, Parkinson's disease, Stroke, Genetics

## Abstract

Brain diseases have complex patterns of genetic and environmental risk factors, and better understanding of these risks is required for more effective prevention strategies. Participants of the Dutch Brain Research Registry provided detailed information on family structure and occurrence of brain diseases. A total of 12,040 participants (73% female, aged 64.9 ± 11 years) provided information on 101,379 family members (53% female, aged 62 ± 25 years). We estimated heritability (*h*^2^) of the nine most common brain diseases using polygenic modeling in SOLAR and assessed variations in *h*^2^ through bootstrapping; Alzheimer’s disease (AD) (*h*^2^ = 73, range 53–86, *P*_fdr_ < 0.001), ALS (*h*^2^ = 72, range 10–98, *P*_fdr_ = 0.030), frontotemporal dementia (FTD) (*h*^2^ = 48, range 0–97, *P*_fdr_ = 0.132), vascular dementia (VaD) (*h*^2^ = 41, range 7–64, *P* = 0.003), Lewy Body dementia (*h*^2^ = 34, range 0–58, *P* = 0.132), iCVA (*h*^2^ = 27, 6–59, *P*_fdr_ = 0.013), hCVA (*h*^2^ = 29, 8–57, *P*_fdr_ = 0.007), Parkinson’s disease (PD) (*h*^2^ = 38, 6–66, *P*_fdr_ = 0.013), and multiple sclerosis (*h*^2^ = 10, 10–97, *P*_fdr_ < 0.001). Shared environmental effects could be estimated for AD (*c*^2^ = 5.8%, *P*_fdr_ = 0.011), VaD (*c*^2^ = 9.0%, *P*_fdr_ = 0.021), FTD (*c*^2^ = 9.7%, *P*_fdr_ = 0.33), iCVA (*c*^2^ = 15.9%, *P*_fdr_ < 0.001), hCVA (*c*^2^ = 14.9%, *P*_fdr_ = 0.005), and PD (*c*^2^ = 7.5%, *P*_fdr_ = 0.25). These findings underscore the significance of genetic contribution to most brain diseases and the important role of shared environments in AD and vascular-related conditions, highlighting initiatives to mitigate modifiable risk factors.

## Introduction

Brain diseases, including Alzheimer’s disease (AD), other dementias, Parkinson’s disease (PD), multiple sclerosis (MS), amyotrophic lateral sclerosis (ALS) and stroke, are among the leading causes of death and disability worldwide^1^. These disorders have a complex pattern of genetic and environmental risk factors^2–5^, and understanding these risks can improve prevention and treatment. A disease’s genetic contribution, or heritability (*h*^2^), represents the proportion of variance explained by shared genetic factors such as common and rare genetic variation^6–8^.

Heritability is estimated using mixed-effects models that quantify genetic and environmental variance, and has traditionally relied on nuclear families, small pedigrees, or twin studies^9^. Previous twin and pedigree studies estimated heritability for AD at 79% (range 67–88)^10^, ALS 61% (range 38–78)^11^, MS 50% (range 39–61)^12^, PD 39% (range 28–44)^2^, and stroke death 32% (range 4–47)^13^. While heritability estimates for more uncommon brain diseases, such as frontotemporal dementia (FTD), Lewy body dementia (DLB), and vascular dementia (VaD) remain limited, recent large-scale genomic studies have begun to address this gap^14^. Nonetheless, conventional heritability studies, often based on small sample sizes, typically fail to capture enough cases to reach reliable estimates. Recently, large biobanks and national registers have emerged as a key method to identify genetic and environmental influences on brain diseases. This enables the study of heritability across a wide range of brain diseases within a large population-based sample in a single study^15^.

Next to estimates of heritability, complex families can be used to estimate shared environmental factors to brain diseases, such as shared lifestyle and household conditions^9^. Environmental exposures, including air pollution, pesticides, and heavy metals, are often shared by family members and may be preventable risks factors for neurodegenerative diseases^3,16,17^. Insights in which diseases are determined by shared environmental factors can inform public health policies aimed at reducing disease incidence^16,18^.

Here, we estimate heritability and shared environmental variance (*c*^2^) for nine brain diseases (including FTD, DLB and VAD) using family structures and history of thousands of participants of a population-based research registry.

## Results

### Subject characteristics

Table 1 presents the subject characteristics. The studied cohort comprised 101,379 individuals of whom 53% was female, with a mean age at assessment of 57.6 ± 20 years and a mean age at death of 76.7 ± 15 years. AD was the most frequently reported condition, affecting 8,149 individuals (8.0% of cohort), with a mean age at onset of 77 ± 8.8 years. This was followed by iCVA in 2436 individuals (2.4%, mean age at onset 69 ± 14 years) and hCVA in 2165 individuals (2.1%, mean age at onset 69 ± 15 years). The conditions with the lowest frequencies were MS, affecting 223 individuals (0.2%, mean age at onset 38 ± 12 years); DLB, affecting 192 individuals (0.2%, mean age at onset 76 ± 8.6 years); and ALS, affecting 71 individuals (0.1%, mean age at onset 64 ± 11 years).

**Table 1:** Demographic and disease characteristics of the cohort used for analyses

Subjects				
Total: Final analysis cohort	101,379 (100)			
Female	53,958 (53)			
Age alive^a^, *mean* *±* *SD*	57.6 ± 20			
Died^b^	51,296 (51)			
Died under 5 years old	6280 (6)			
Died 5 years and older	44,555 (44)			
Age at death (5 + )^c^, *mean* *±* *SD*	76.7 ± 15			
Brain diseases	*N* cases (%)	*N* families (%)	Age at onset *(mean* *±* *SD)*	Range in onset, years
Alzheimer’s disease	8149 (8)	5195 (43)	77.0 ± 8.8	[38–101]
Vascular dementia	2040 (2)	1764 (15)	78.2 ± 8.2	[40–100]
Frontotemporal dementia	244 (0.2)	218 (1.8)	71.3 ± 11	[40–92]
Lewy Body dementia	192 (0.2)	189 (1.6)	75.7 ± 8.6	[47–97]
Ischemic CVA	2436 (2)	1977 (16)	68.6 ± 13.9	[10–100]
Hemorrhagic CVA	2165 (2)	1765 (15)	69.1 ± 15	[11–104]
Parkinson’s disease	1091 (1)	985 (8.2)	67.8 ± 11	[25–95]
Multiple sclerosis	223 (0.2)	203 (1.7)	37.7 ± 11.7	[8–67]
Amyotrophic lateral sclerosis	71 (0.1)	67 (0.6)	63.8 ± 11.3	[32–85]

N (%) unless otherwise specified. Alzheimer’s disease is grouped with dementia of unspecified etiology.

Total n: a = 49,553; b = 100,914; c = 50,835.

*CVA* cardiovascular accident, *NA* not available, *SD* standard deviation.

### Heritability estimates

**Fig. 1:**
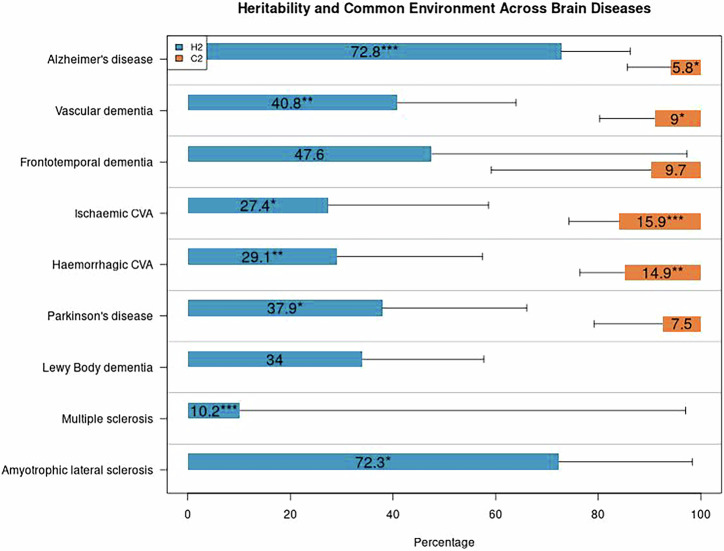
Estimates of genetic (*h*^*2*^) and shared (*c*^*2*^) environmental effects across brain diseases. *p < 0.05, **p < 0.01, ***p < 0.001. *P* values were adjusted using the Benjamini-Hochberg false discovery rate (FDR) correction. For DLB, MS, and ALS, the number of affected cases was below 225, and thus polygenic models did not include a shared environmental effect.

We list the diseases from high to low heritability. The heritability of AD, including dementia of unspecified etiology, was estimated to be 72.8%, ranging between 53 and 86 (95% confidence interval (*CI*), *P*_fdr_ < 0.001) (Fig. 1, Supplementary Table 3). Similarly, the heritability of ALS was estimated to be 72.3% (*CI* 10–98, *P*_fdr_ = 0.030), followed by FTD, which was estimated to be 47.6% (*CI* 0–97, *P*_fdr_ = 0.132), and the heritability of VaD was estimated to be 40.8% (*CI* 7–64, *P*_fdr_ = 0.003). The heritability of PD was estimated to be 37.9% (*CI* 6–66, *P*_fdr_ = 0.013), and 34.0% for DLB (*CI* 0–58, *P*_fdr_ = 0.132). The heritability of hCVA was modest and estimated to be 29.1% (*CI* 8–57, *P*_fdr_ = 0.007), while the heritability of iCVA was estimated 27.4% (*CI* 6–59, *P*_fdr_ = 0.013). The heritability for MS was estimated to be the lowest of all diseases: 10.2% (*CI* 10–97, *P*_fdr_ < 0.001).

### Shared environmental effect

Shared environmental effect estimates on disease prevalence among family members ranged from 5.8 to 15.9% (Fig. 1, Supplementary Table 3). Among the different dementia types, AD showed a small shared environmental effect of 5.8% (*CI* 1–14, *P*_fdr_ = 0.011). FTD demonstrated a modest, but non-significant, shared environmental effect of 9.7% (*CI* 0–41, *P*_fdr_ = 0.33), as well as VaD of 9.0% (*CI* 1–20, *P*_fdr_ = 0.021). Among other brain diseases, iCVA showed the highest shared environmental effect of 15.9% (*CI* 1–26, *P*_fdr_ < 0.001), followed closely by hCVA of 14.9% (*CI* 1–24, *P*_fdr_ = 0.005). PD demonstrated a modest shared environmental effect of 7.5% (*CI* 0–21, *P*_fdr_ = 0.25). Note that for DLB, MS, and ALS the shared environmental effect could not be calculated due to limited power (fewer than 225 affected cases).

## Discussion

We performed genetic analyses using a large extended family design to demonstrate significant heritability of common brain diseases including: AD (including dementia of unspecified etiology), VaD, ischemic CVA, hemorrhagic CVA, PD, MS, ALS. Notably, we presented the first estimates for FTD, DLB, and VaD, as prior twin studies lacked such estimates. Next to genetics and albeit a smaller effect, a significant portion of variability within families was explained by shared environmental effects for AD, VaD, iCVA, and hCVA – all diseases known to be strongly associated with vascular risk factors^16^.

**Fig. 2:**
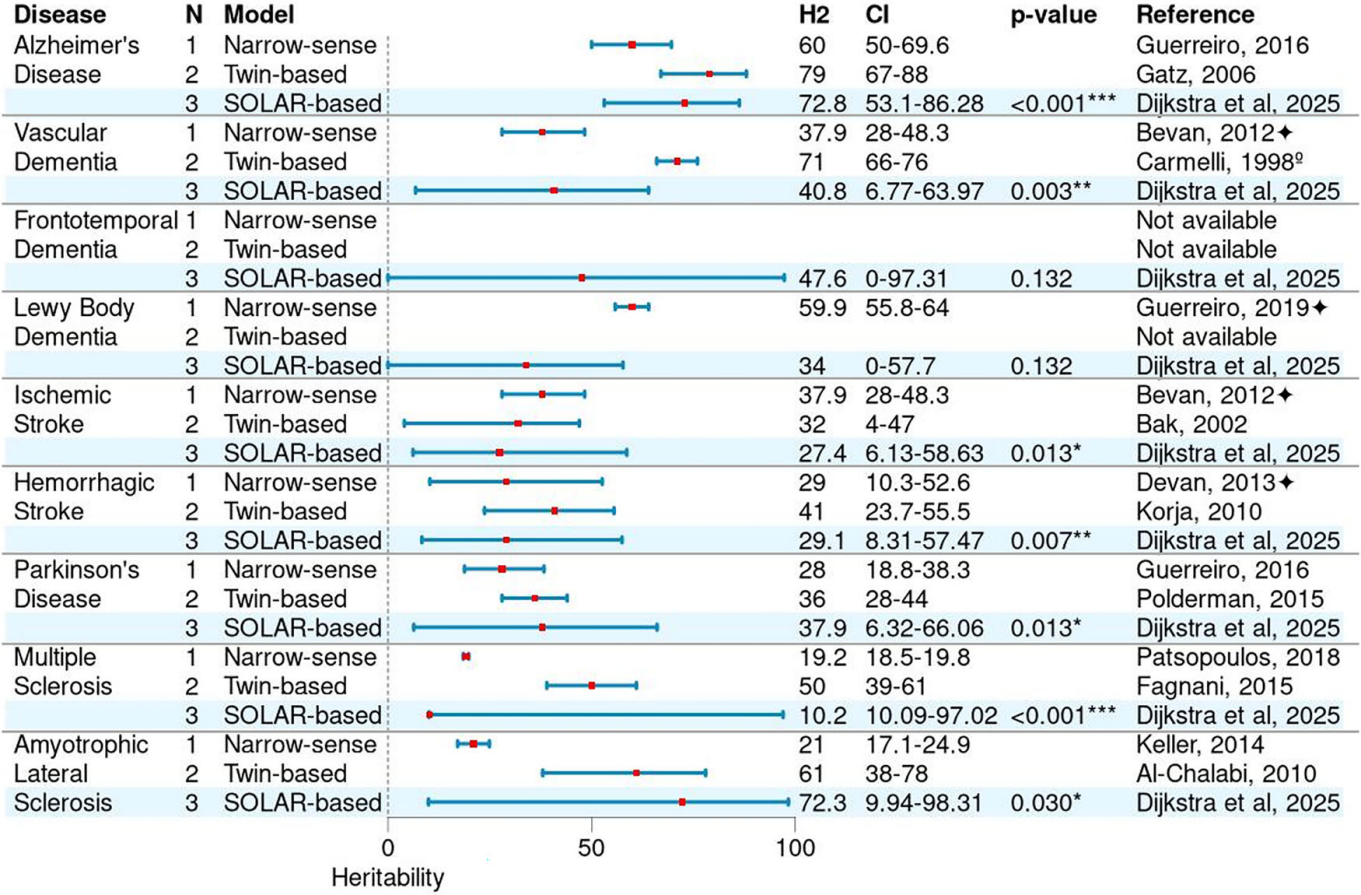
Heritability estimates of brain diseases based on narrow-sense, twin-based, and SOLAR-based heritability models. *p < 0.05, **p < 0.01, ***p < 0.001. *P* values were adjusted using the Benjamini-Hochberg false discovery rate (FDR) correction. CI = 95% confidence interval, H2 heritability estimate, *N* = number. Alzheimer’s disease is a combined category with dementia of unspecified etiology. Narrow-sense models are based on genome-wide association studies or genome-wide complex trait analysis✦. °Interpret with caution. In the absence of direct twin studies for vascular dementia, we used a twin study on the heritability of white matter hyperintensities (WMH) as proxy because WMHs are strongly associated with small-vessel disease, a major contributor to vascular dementia. See the Supplementary Discussion for further interpretation of our findings.

We estimated heritability, which represents the total genetic contribution to a trait, encompassing both additive effects and more complex genetic interactions^19,20^. Our heritability estimates from extended families were compared to those from previous twin studies (Fig. 2, Supplementary Discussion). At large, we confirmed the strong genetic component in AD^10^ and ALS^11^, as well as moderate genetic contributions in PD^2^ and stroke^13^. However, our findings indicated a smaller genetic component in MS (10.2%, range 10–97), although confidence intervals overlapped with prior estimates (range 39–61%)^12^. This discrepancy may be attributed to the low prevalence of MS within families in our cohort (*n* = 223 cases in *n* = 203 families), leading to a less precise estimate. Additionally, differences between twin-based and SOLAR-based heritability estimates may arise from misclassification of diseases, underreporting in older generations and differences in statistical power between twin-based studies and complex pedigree approaches. These differences may have led to either an underestimation or overestimation of heritability in our work compared to twin-based studies.

The rarity of FTD, DLB and VaD precluded previous estimation of heritability using twin studies, such that we compared our heritability with other reported measures of familiarity. For FTD, we report moderate genetic contributions (47.6%, range 0–97), which align with the observation that up to 40–50% of FTD patients report a family history of dementia^21^, supporting the prominent role of hereditary factors in FTD’s etiology. Beyond genetic causes, while familial mutations account for around one-fifth of FTD cases, the majority of FTD appears to result from a complex interplay of multiple genetic and environmental factors^22^. For DLB, we report a moderate genetic contribution (34.0%, range 0–58), in line with the findings of a previous genome-wide complex trait analysis (59.9%, *SE* 2.1)^23^. For VaD, heritability estimates were moderate (40.8%, range 7–64), which is in line with a genome-wide complex trait analysis on large artery, cardioembolic, and small vessel stroke (37.9%, *SE* 5.2)^24^, and partly in line with a twin study on white matter hyperintensity volume (71%, range 66–76)^25^. As small-vessel disease is a major contributor to vascular cognitive decline and is strongly associated with white matter hyperintensities, this further supports a genetic component in VaD’s pathophysiology.

We estimated heritability using bootstrapping, where each combination of families in a bootstrap sample may yield a different heritability estimate. Families with autosomal dominant inheritance patterns (where heritability is estimated at 100%) are combined with families exhibiting sporadic disease occurrence. The distribution of heritability across bootstrap samples provides insight into the underlying genetic architecture of the diseases. Heritability estimates followed a normal distribution for AD, VaD, iCVA, hCVA, and PD (Supplementary Fig. 2), which suggests a predominantly polygenic basis for these diseases with minimal evidence for monogenic inheritance. Conversely, heritability distributions observed for the rarer diseases—i.e., FTD, DLB, MS, and ALS—did not follow a normal distribution, which suggest a mixture of sporadic cases and potential monogenic families. These patterns resulted in relatively wide confidence intervals, and in some cases, bimodal distributions (e.g., for MS and ALS). Important to note is that the likelihood approach tends to underestimate the monogenic component of variance because it assumes a continuous distribution of genetic effects and may not fully capture the discrete, large-effect contributions of rare monogenic variants^20^. Nonetheless, these patterns suggest that while common genetic factors contribute to disease risk, rare high-impact variants may explain some of the observed variability, particularly in the less frequent conditions.

While genetic factors dominated most diseases, shared environmental influences also played a notable role, particularly in VaD and stroke. The strongest shared environmental effects were observed for iCVA (15.9%) and hCVA (14.9%), with more modest contributions for VaD (9.0%) and AD, including dementia of unspecified etiology (5.8%). These findings are partly consistent with existing literature, which reports environmental effects common to the whole family for stroke at 3–4% and AD at 3–6%^18^.

For stroke, known modifiable risk factors—including shared and unique environmental effects—include smoking, physical inactivity, diabetes, and exposure to air pollution^26^. For all-cause dementia, the *Lancet Commission* also identified these modifiable dementia risk factors, as well as low education, depression, and infrequent social contact, among others^16^. One study by Low et al. linked these modifiable risk factors to an increased risk of small vessel disease in midlife, underscoring the need for preventative measures as early as midlife^27^.

On the one hand, our study benefits from the use of a large, community-based volunteer cohort, which offers an alternative for estimating heritability where case ascertainment is often challenging or unfeasible^19,20,28^. On the other hand, self-reported data as obtained in our cohort may involve recall bias and potential misdiagnoses that may impact accuracy. We cannot exclude family duplication in the dataset, which may have inflated or deflated heritability estimates. Due to our study design we are not able to assess the effects of other factors that affect heritability, such as spousal correlation/assortative mating, maternal effects^9^, population stratification, and epistasis^6^.

The genetic components in AD, VaD, iCVA, hCVA, PD, MS, and ALS ranged from 10 to 73%, emphasizing the need for continued genetic research to identify risk genes and pathways, and potential therapeutic targets. The robust shared environmental effect in vascular-related brain diseases underscores the importance of addressing familial risk factors through public health initiatives.

## Methods

### Dutch brain research registry

Data on family structure and the presence of brain diseases were collected via a digital survey distributed to all subscribers of the Dutch Brain Research Registry^29^. This online platform is open for people (minimum age of 18) interested in brain research. Registrants received an invitation via email and after showing interest informed consent was obtained through an electronic procedure. We requested information on first- and second-degree relatives, including the occurrence and age at onset of 15 brain diseases (Supplementary Table 1). The survey adapted interactively based on participants’ responses. Data was collected from May 2023 to January 2024. Ethical approval was granted by the medical ethics committee of the VU University Medical Center (Amsterdam UMC).

### Participant selection and family data scope

**Fig. 3:**
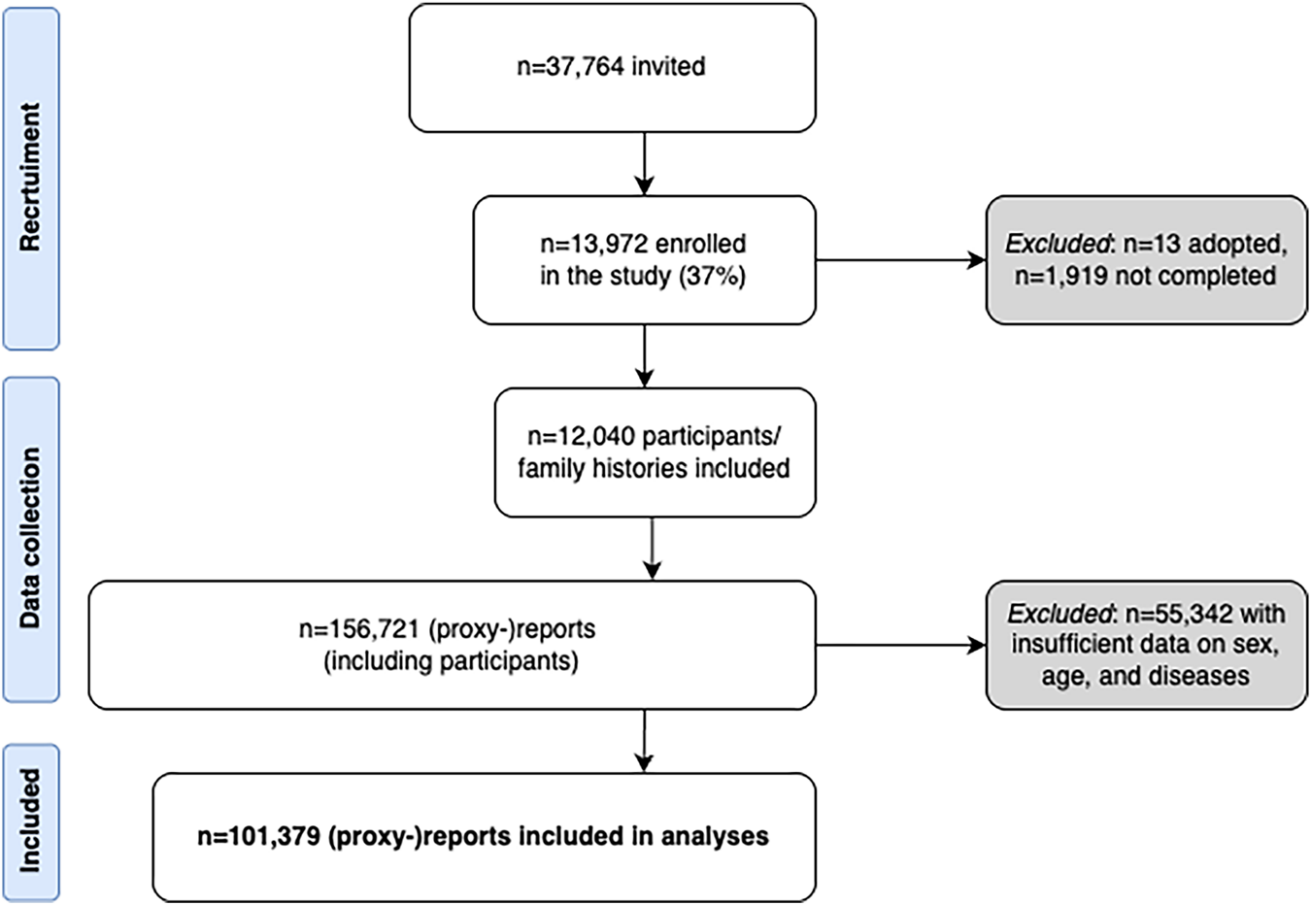
Recruitment flowchart of the Dutch Brain Research Registry, data collection, and final analysis cohort. The flowchart shows participants recruited through the digital survey of the Dutch Brain Research Registry. Data on family structure and the presence of brain diseases (‘family histories’) were collected for both participants and their family members (‘proxy reports’). Reports were included in the analyses if sufficient information was available on sex, age, and disease status. The final dataset included *n* = 12,040 family histories.

Of 37,764 invited individuals, 13,972 enrolled (37% response rate, Fig. 3). Thirteen adoptees and 1919 incomplete surveys were excluded, yielding a final cohort of 12,040 participants Most participants identified as Dutch (97%), with a median education level of 6 (Verhage scale, range 1–7)^30^. All descriptive statistics are in Supplementary Table 1. Participants reported on 156,721 family members (descriptive statistics in Supplementary Fig. 1). Inclusion criteria required complete data on age, sex, and disease status, resulting in 101,379 individuals for subsequent analysis.

### Data analysis

AD (*n* = 4399) and ‘dementia of unspecified etiology’ (*n* = 3750) were grouped under AD. Participants were observed to report either AD (*n* = 2538) or dementia of unspecified etiology (*n* = 2405), but rarely both within the same family (*n* = 642 families, 11%). Thus, the two phenotypes were nearly mutually exclusive at the family level. Given that AD is the most common form of dementia, we combined these categories to more accurately capture familial aggregation. Rare diseases (50 cases or less) were excluded, including Huntingon’s disease, spinocerebellar ataxia, progressive supranuclear palsy, corticobasal syndrome, and Creutzfeldt-Jakob’s disease. Cases with dementia onset before age 20 (*n* = 6; 0.006% of the cohort), and cardiovascular accidents before age 10 (*n* = 22) were excluded, as the biological mechanisms of these conditions are not considered to be associated with age-related neurodegeneration^2,16^.

### Statistical analyses

R (v4.3.0) was used to reconstruct pedigrees with custom scripts (https://github.com/holstegelab/pedigrees) and to analyze data. We used the Linux Sequential Oligogenic Linkage Analysis Routines (SOLAR-Eclipse) Eclipse General v9.0.1 (https://www.nitrc.org/projects/se_linux) to estimate heritability^19,20^. SOLAR-Eclipse is an imaging genetics analyses software and uses an iterative maximum-likelihood model. In twin and sibling research, a strong correlation has been observed between SOLAR and other methods used to estimate heritability^28^. The pedigree-based design assumes additive genetic effects that are summarized as coefficients of relatedness in a kinship matrix^31^. The percentage of shared genetics follows the principles of inheritance within families. For example, parent-child relationships and full siblings share an average of 50% of their genetic factors, grandparent-child relationships and half-siblings share 25%, while unrelated partners share 0%. SOLAR incorporates this kinship matrix into the following formula^28^:

$$\varOmega =2\cdot \varPhi \cdot {s}_{g}^{2}+H\cdot {s}_{c}^{2}+I\cdot {s}_{e}^{2}$$

where the multivariate normal covariance matrix for a pedigree ($\varOmega$) is calculated by the sum of pairwise kinship coefficients ($\varPhi$) times the genetic phenotypic variance (${s}_{g}^{2}$), and $I$ as an identity matrix to calculate the variance due to a unique environment (${s}_{e}^{2}$). In family-based designs the variance due to a common environment (${s}_{c}^{2}$) may be added in which $H$ is the shared environment matrix.

Polygenic models were computed for all diseases and adjusted for sex, age, age × sex interaction, squared age (age^2^), and age^2^ × sex interaction. We included both linear (age) and non-linear (age^2^) terms to account for non-linear effects of age on the outcome, as age-related processes are often not strictly linear across the lifespan. Additionally, interaction terms between age and sex (age × sex, age^2^ × sex) were included to adjust for potential sex differences. Age was determined per disease, prioritizing the reported symptom onset, followed by age at assessment or age at death. To estimate the common environmental variance (*c*^2^), a household component was included in the polygenic models. Each family was considered to share a household. For diseases that affected less than 225 individuals (i.e., DLB, MS, ALS; Supplementary Table 2), a household component could not be included due to failure of the models to converge (too little power).

The SOLAR program was designed to determine environmental variance attributable to household components in samples comprising a maximum of 32,000 individuals. As our sample was over 100 thousand individuals, we addressed this using bootstrapping. We used 3000 bootstrap samples, each consisting of 2000 families, to calculate *h*^2^, *c*^2^, and *e*^2^. For AD, given its high prevalence—with families having between 1 and 14 affected members (Supplementary Table 2)—we reduced the sample size to 1000 families per bootstrap sample. This resulted in a total of 2890 successful bootstrap samples after correcting for an error related to the high prevalence. All bootstrap sets of families were randomly selected with replacement. To visualize the distribution of heritability estimates, we plotted distribution and density plots of the bootstrap models of all brain diseases.

To assess robustness, we also computed models without household components. As this analysis did not impose the threshold of 32,000 individuals, we selected 1000 bootstrap samples across all 12,040 families to estimate *h*^2^ and *e*^2^ and their confidence intervals.

## Supplementary information

Supplementary Information

## Data Availability

The data supporting the findings of this study can be accessed upon reasonable request to the corresponding author. The underlying code for this study is not publicly available but may be made available to qualified researchers on reasonable request from the corresponding author.
