# Supplementary material for: Heritability and shared environmental effects of brain diseases in 12,040 extended families
Source: NPJ Dement. 2025 Nov 3;1(1):34. doi: 10.1038/s44400-025-00030-2 (PMC12583135; doi:10.1038/s44400-025-00030-2)
Supplement: Supplementary file 1 — Supplementary Information [file 44400_2025_30_MOESM1_ESM.pdf]

SUPPLEMENTARY MATERIAL

Supplementary Table 1. Demographic and disease characteristics of participants, total cohort, and cohort used for heritability analyses.

	Participants	Total cohort	Analysis cohort
Total	12,040 (100)	156,721 (100)	101,379 (65)
Female[a]	8,812 (73)	73,519 (51)	53,958 (53)
Age alive[b], *mean ±SD*	64.9 ±11	57.3 ±20	57.6 ±20
Died[c]	0	73,899 (57)	51,296 (51)
Died under 5 years old	0	8,921 (7)	6,280 (6)
Died 5 years and older	0	48,956 (37)	44,555 (44)
Age at death (5+)[d], *mean ±SD*	NA	76.3 ±15	76.7 ±15
Ethnicity: Dutch	11,659 (97)	NA	NA
Education[e]**, *median [IQR]*	6 [5-6]	NA	NA
Brain diseases[g]			
Alzheimer's disease	53 (0.44)	9,632 (8.3)	8,149 (8.0)
Vascular dementia	18 (0.15)	2,191 (1.9)	2,040 (2.0)
Frontotemporal dementia	7 (0.06)	260 (0.22)	244 (0.24)
Lewy Body dementia	3 (0.02)	200 (0.17)	192 (0.19)
Ischemic CVA	419 (3.5)	2,622 (2.3)	2,436 (2.4)
Hemorrhagic CVA	144 (1.2)	2,390 (2.1)	2,165 (2.1)
Parkinson's disease	54 (0.45)	1,249 (1.1)	1,091 (1.1)
Multiple sclerosis	77 (0.64)	238 (0.2)	223 (0.22)
Amyotrophic lateral sclerosis	1 (0.01)	79 (0.07)	71 (0.07)
Huntington's disease	3 (0.02)	41 (0.04)	28 (0.03)
Spinocerebellar ataxia	5 (0.04)	31 (0.03)	26 (0.03)
Progressive supranuclear palsy	3 (0.02)	18 (0.02)	15 (0.01)
Corticobasal syndrome	0	13 (0.01)	12 (0.01)
Creutzfeldt-Jakob's disease	0	13 (0.01)	11 (0.01)

N (%) unless otherwise specified.

*p-value <0.05. **according to the Dutch Verhage scale (range 1-7).

Total n: a = 12040, 145178, 101379; b = 12027, 53610, 49553; c = 12040, 130661, 100914; d = 0, 57877, 50835; e = 12021, 0, 0.

Abbreviations: CVA = Cardiovascular Accident; IQR = Interquartile Range; NA = Not Available; SD = Standard Deviation.

Supplementary Table 2. Family characteristics of cohort used in heritability analyses.

	**N of affected individuals**	**N of affected families**	**Range of affected family members within family**
Alzheimer's disease	8,149	5,585	1-13
Vascular dementia	2,040	1,823	1-10
Frontotemporal dementia	244	228	1-4
Ischemic CVA	2,436	2,070	1-6
Hemorrhagic CVA	2,165	1,890	1-8
Parkinson's disease	1,091	1,108	1-5
Lewy Body dementia	192	197	1-3
Multiple sclerosis	223	218	1-4
Amyotrophic lateral sclerosis	71	72	1-4

N (%) unless otherwise specified.

Supplementary Table 3. Estimates of genetic (h^2), common (c^2), and unique environmental (e^2) effects across brain diseases.

	h^2 (%)	CI (%)	*p (*FDR)	c^2 (%)	CI (%)	*p (*FDR)	e^2 (%)
AD	72.8	[53, 86]	<0.001***	5.8	[1, 14]	0.0111*	22.3
VaD	40.8	[7, 64]	0.0030**	9.0	[1, 20]	0.0205*	51.7
FTD	47.6	[0, 97]	0.1323	9.7	[0, 41]	0.3327	37.0
iCVA	27.4	[6, 59]	0.0130*	15.9	[1, 26]	<0.001***	56.3
hCVA	29.1	[8, 57]	0.0068**	14.9	[1, 24]	0.0050**	56.0
PD	37.9	[6, 66]	0.0130*	7.5	[0, 21]	0.2500	54.3
LBD	34.0	[0, 58]	0.1323	NA	NA	NA	66.0
MS	10.2	[10, 97]	<0.001***	NA	NA	NA	89.8
ALS	72.3	[10, 98]	0.030*	NA	NA	NA	27.7

*p<0.05, **p<0.01, ***p<0.001. P-values were adjusted using the Benjamini-Hochberg false discovery rate (FDR) correction.

Note. AD = Alzheimer's Disease; ALS = Amyotropic Lateral Sclerosis; CVA = Cardiovascular Accident; CI = 95% confidence interval; FTD = Frontotemporal Dementia; H = Hemorrhagic; I = Ischemic; LBD = Lewy Body Dementia; MS = Multiple Sclerosis; NA = Not Available; PD = Parkinson's Disease; SE = Standard Error; VaD = Vascular Dementia.

Number of bootstraps for brain diseases: 2890, 3000, 3000, 3000, 3000, 3000, 1000, 1000, 1000. Bootstrap models included 2,000 randomized families, except for Alzheimer's disease, where 1,000 families were used due to its high disease prevalence. For DLB, MS, and ALS (N affected < 225), bootstrap models included 12,040 randomized families with replacement, and polygenic models did not account for a household effect.

Please note that the permuted estimates from the ACE models are approximately 100, with minor deviations possible due to the nature of the permutation process.

Supplementary Table 4. Estimates of genetic (h^2) and unique (e^2) environmental effects on brain diseases in the total cohort.

	h^2 (%)	CI (%)	p	e^2 (%)
Alzheimer's disease	77.0	[33, 81]	<0.001***	23.0
Vascular dementia	50.3	[36, 57]	<0.001***	49.7
Frontotemporal dementia	76.5	[60, 95]	<0.001***	23.5
Ischemic CVA	54.0	[48, 59]	<0.001***	46.0
Hemorrhagic CVA	53.7	[47, 60]	<0.001***	46.3
Parkinson's disease	53.2	[43, 61]	<0.001***	46.8
Lewy Body dementia	34.0	[0, 58]	0.121	66.0
Multiple sclerosis	10.2	[10, 97]	<0.001***	89.8
Amyotrophic lateral sclerosis	72.3	[10, 98]	0.023*	27.7

*p<0.05, **p<0.01, ***p<0.001.

Note. CI = 95% Confidence Interval; NA = Not Available.

For all brain diseases, polygenic models were conducted for 1,000 bootstrap samples, except for Alzheimer's disease, where 771 bootstraps were used due to its high disease prevalence. Bootstrap models included 12,040 randomized families with replacement.

Supplementary Figure 1. Extended pedigrees from 12,040 families.

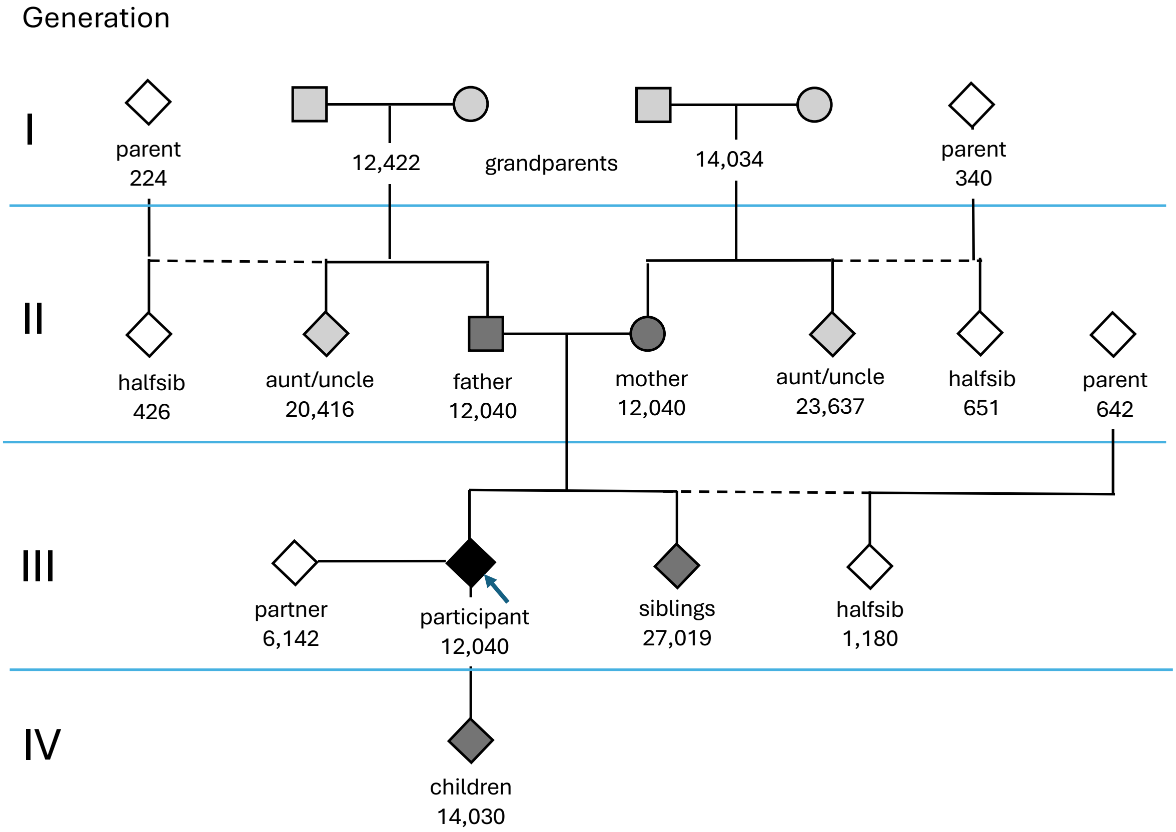

Participants were asked to self-report information on their family members via a digital survey. The numbers indicate the quantity of individuals included from each relationship category.

Supplementary Figure 2. Distribution and density plots of genetic (h^2), common (c^2), and unique environmental (e^2) effects across brain diseases.

Bootstrap models incorporating a shared environmental effect (i.e., AD, FTD, VaD, iCVA, hCVA, PD) were generated using 2,000 randomized families, except for AD, which used 1,000 families due to its high prevalence. In contrast, bootstrap models without a shared environmental effect (i.e., DLB, MS, ALS) were based on a dataset of 12,040 randomized families. Families were randomly selected with replacement for all models to ensure robust estimates.

Alzheimer's disease, including dementia of unclear etiology

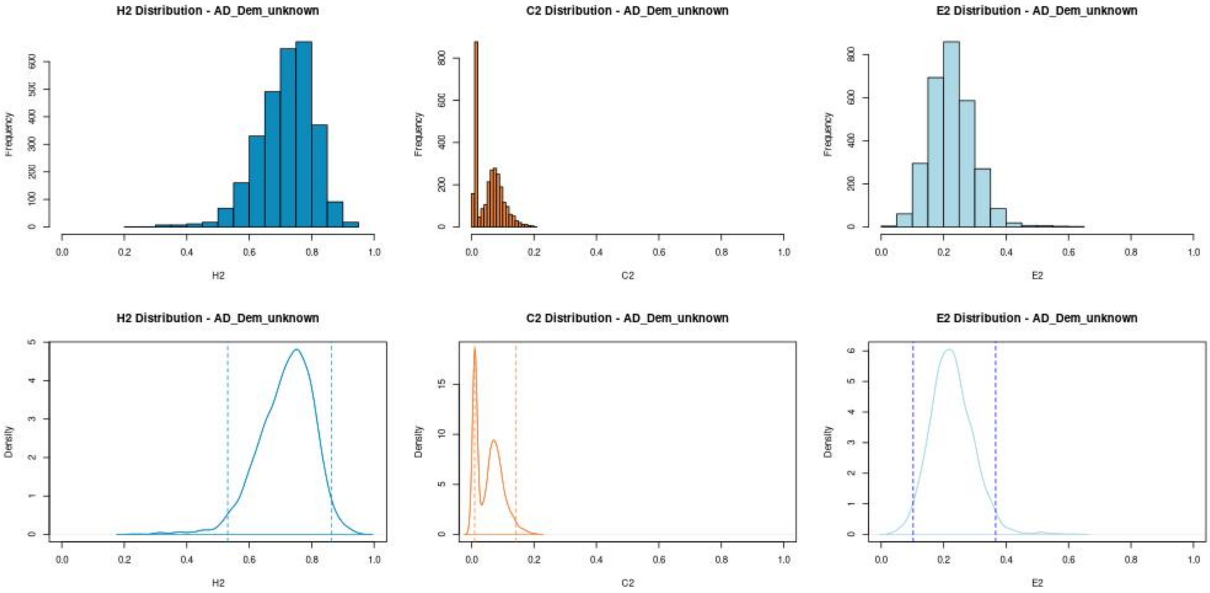

Vascular dementia

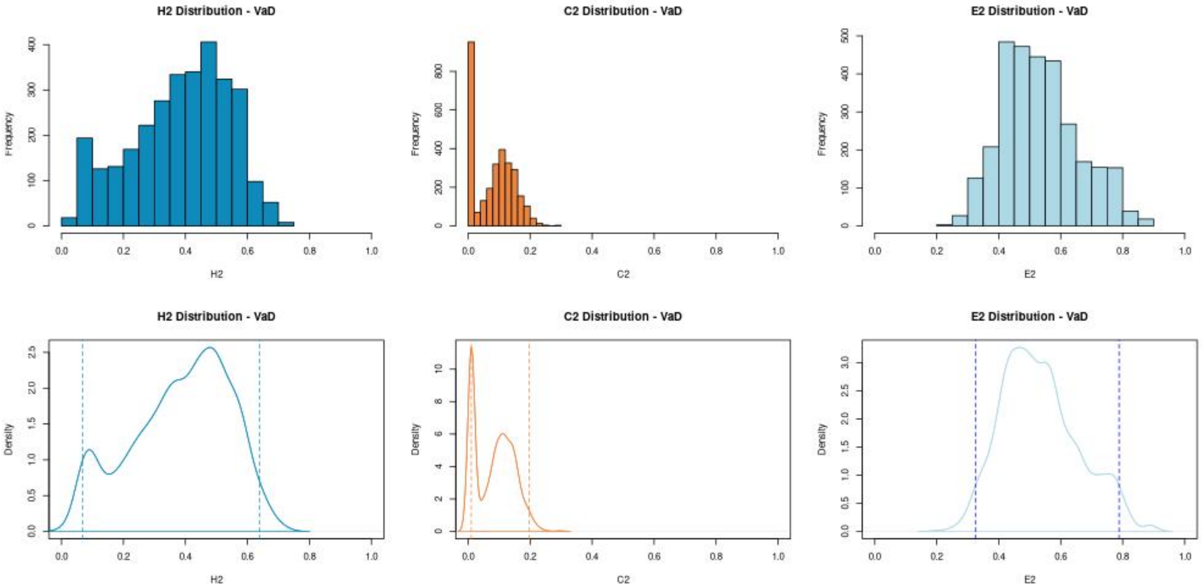

Frontotemporal dementia

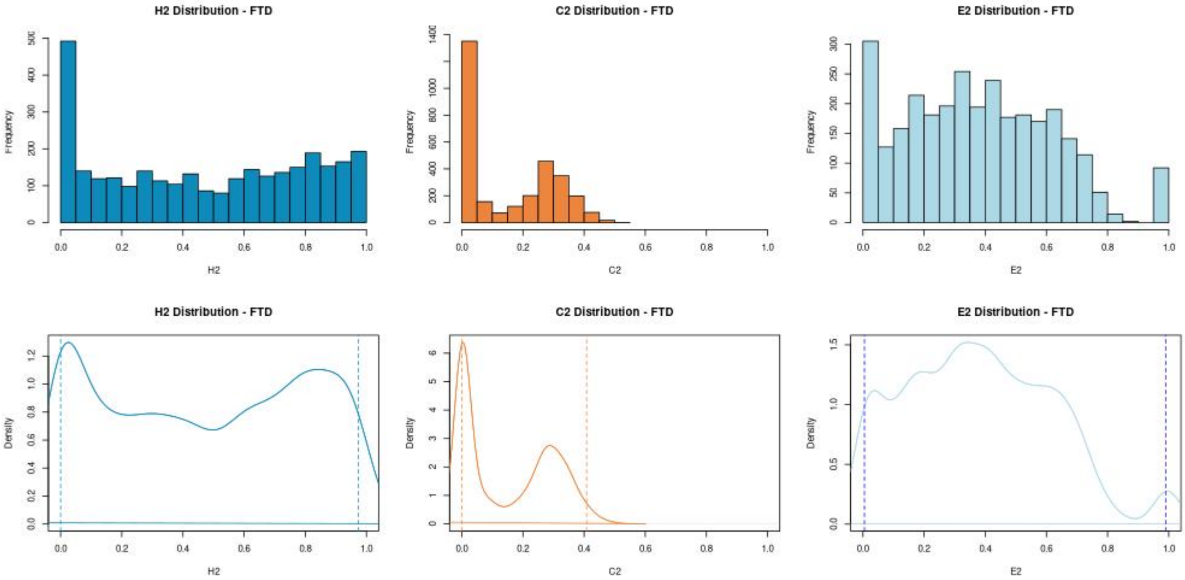

Ischemic stroke

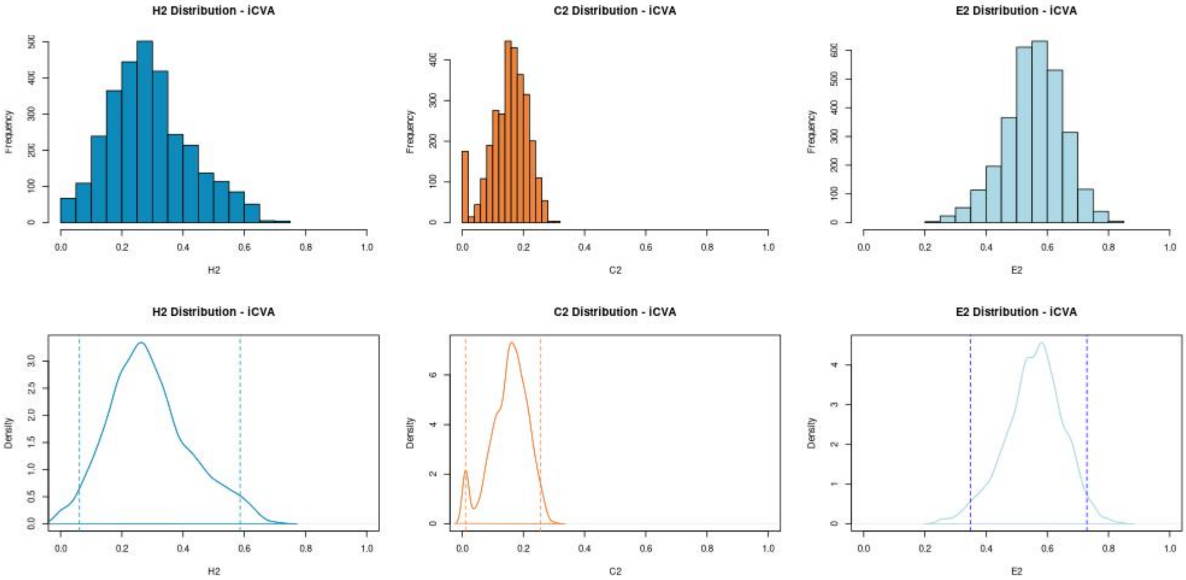

Hemorrhagic stroke

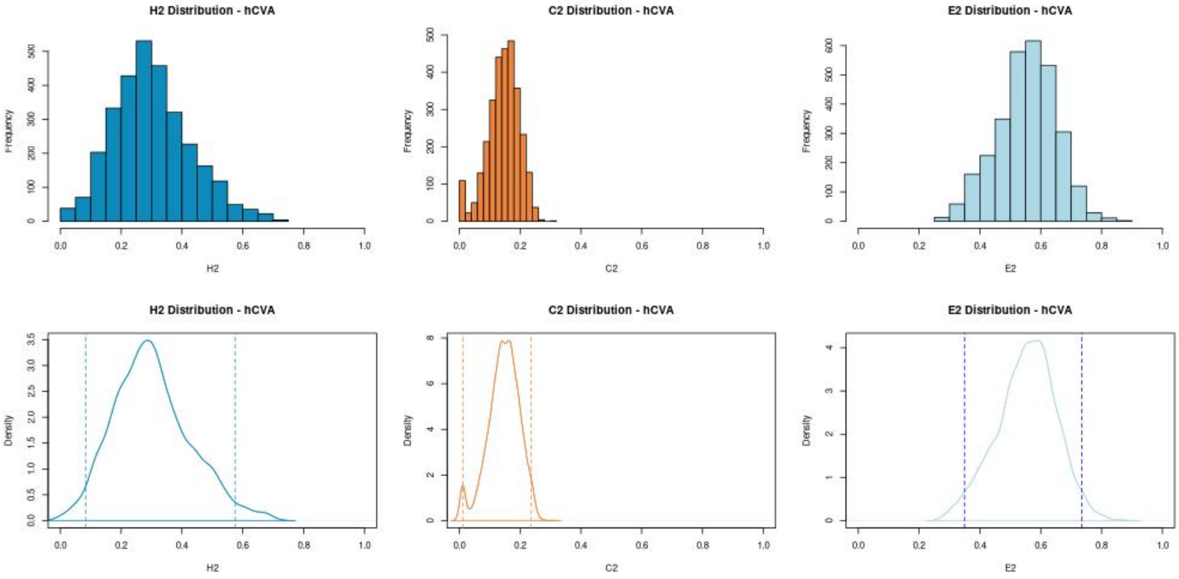

Parkinson's disease

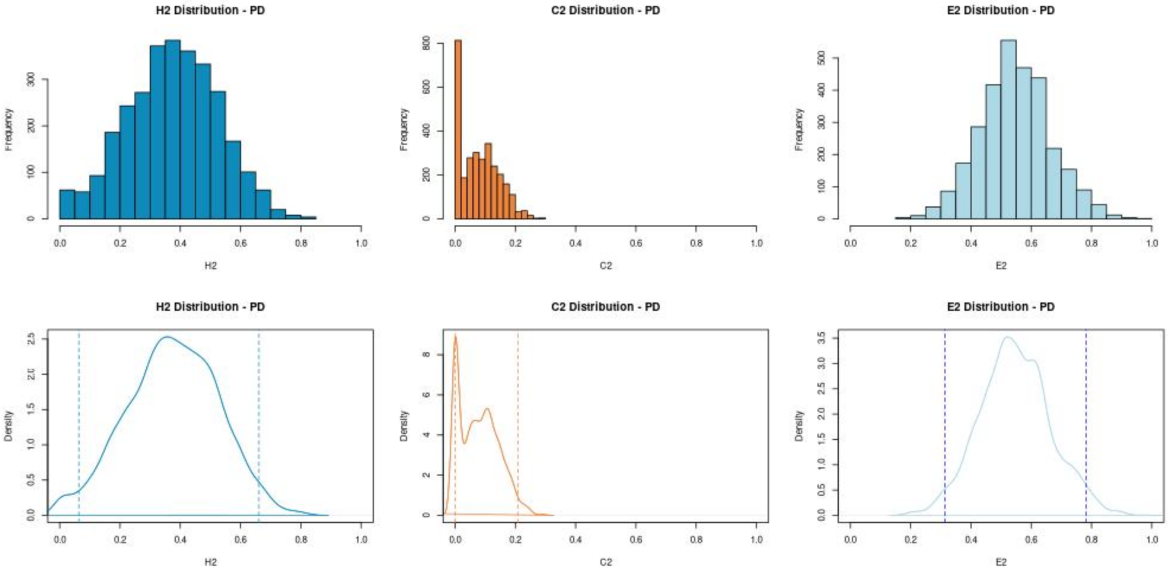

Lewy Body dementia

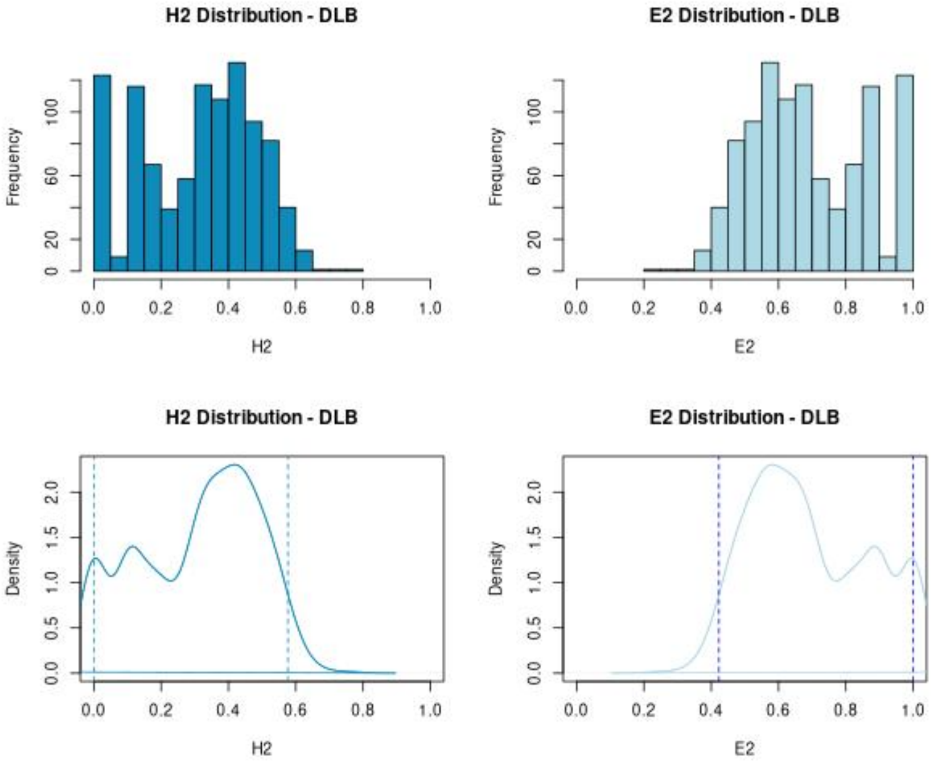

Multiple sclerosis

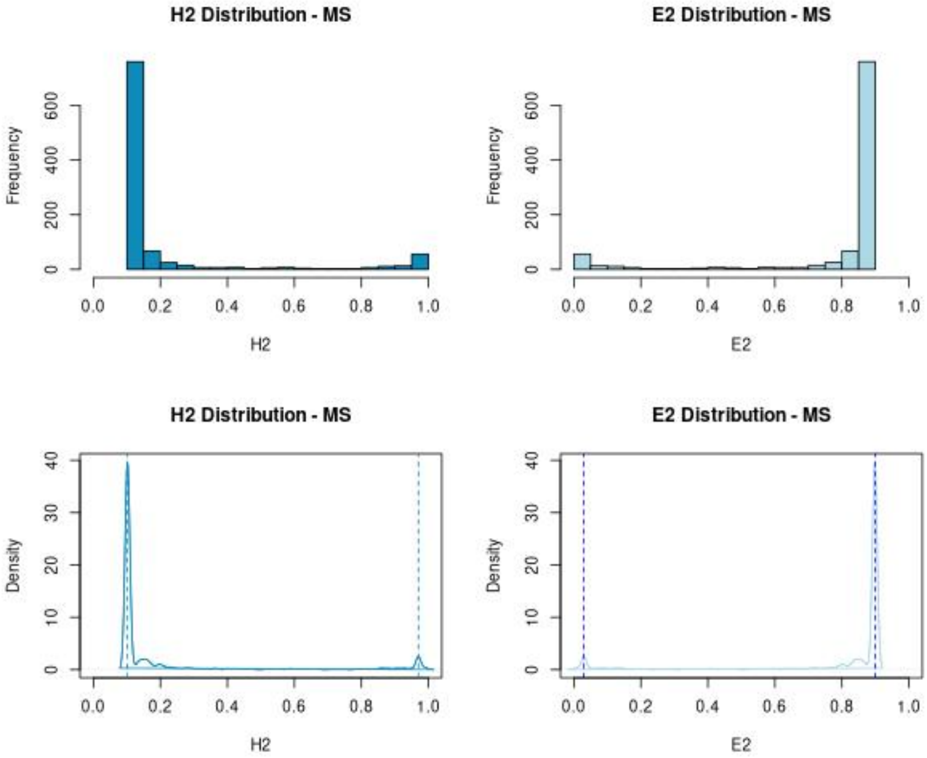

Amyotrophic lateral sclerosis

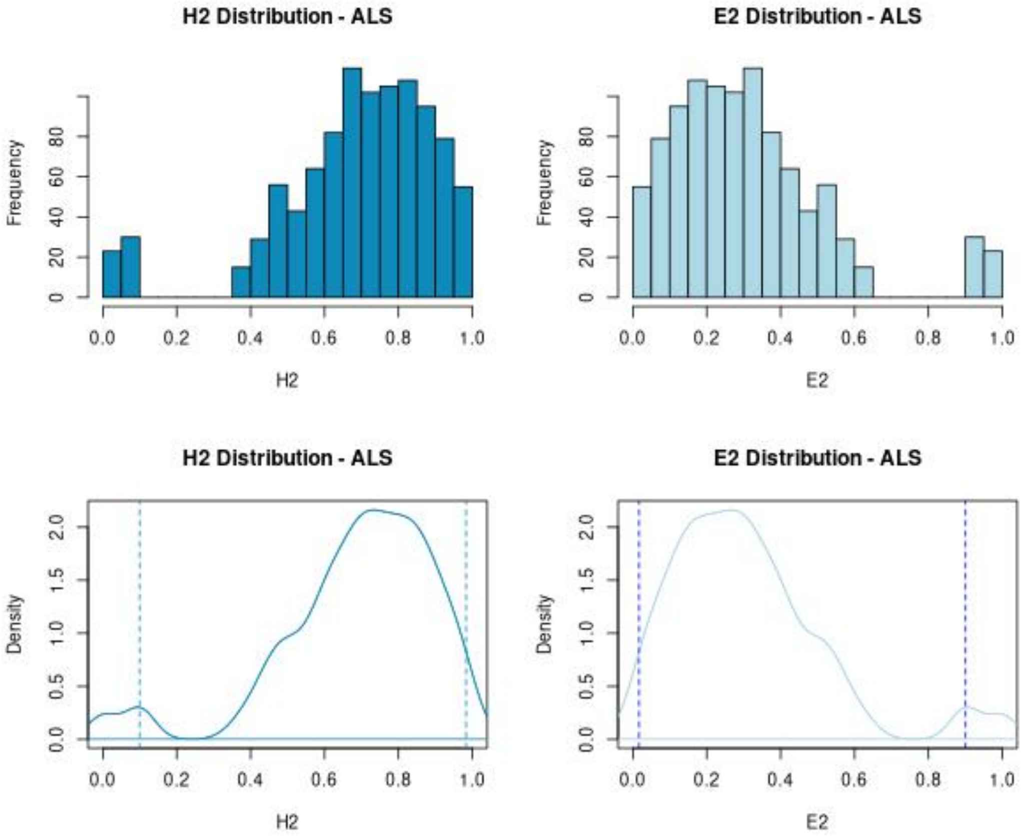

Supplementary Discussion

This supplementary discussion provides a broader perspective on our findings by situating them within the existing body of literature. Specifically, it evaluates our observed broad-sense heritability estimates in the context of twin-based research and estimates of narrow-sense heritability – i.e., single nucleotide polymorphism (SNP)-based – derived from genome-wide association studies (GWAS) and genome-wide complex trait analyses (GCTA). Our results are illustrated in Figure 2.

Alzheimer's disease (AD)

Our findings indicate a high heritability estimate of 72.8% (range 53-86) for AD, reinforcing its well-established genetic basis. This estimate aligns with previous twin studies (range 67-88%)[1] and SNP-based research (range 55-65%),[2] which consistently report high heritability for AD.[3] The robust genetic component in AD underscores the importance of ongoing genetic research to identify specific risk genes and pathways involved in disease development,[4] and justifies the continued focus on potential therapeutic targets.[5]

Frontotemporal dementia (FTD)

FTD exhibited a heritability estimate of 47.6% (range 0-97), marking the first reported estimate of its kind and underscoring a strong genetic influence. Up to 40-50% of FTD patients have a family history of dementia, with about 30% of cases linked to mutations in the *MAPT*, *GRN*, and *C9ORF72* genes.[6] Although GWASs have identified common variants associated with FTD, these explain only a small fraction of its heritability.[7,8] The "missing heritability" may be attributed to the presence of rare variants, structural variants, or polygenic risk beyond GWAS detection. Despite FTD's rarity and the lack of twin studies, the high heritability estimate highlights the need for further genetic research to better understand the specific genetic mechanisms underlying FTD and to develop targeted interventions. Notably, our study also found a 9.7% shared environmental effect – though not significant, the highest among dementias. While familial mutations account for around one-third of FTD cases, the majority of FTD appears to result from a complex interplay of multiple genetic and environmental factors.[9]

Vascular dementia (VaD)

VaD showed a heritability estimate of 40.8% (range 7-64). While no twin studies on VaD have successfully been conducted,[10] one twin study on white matter hyperintensity volume has reported a heritability estimate of 71% (range 66-76),[11] and one genome-wide complex trait analysis on large artery, cardioembolic, and small vessel stroke reported a heritability of 37.9% (*SE* 5.2%).[12] One recent GWAS on VaD reported a heritability of 6.1% (range 3.2-21).[13]

VaD is widely recognized as a heterogeneous disease involving multiple pathological mechanisms.[14,15] For instance, small-vessel disease is a major contributor to vascular cognitive decline and is strongly associated with white matter hyperintensities[11] and ischemic stroke.[13] In terms of genetic heterogeneity, several monogenic forms of vascular impairment have been linked to mutations in genes such as *NOTCH3, HTRA1,* and *FOXF2.*[14,15] GWAS studies have identified many common loci associated with vascular health, including genes related to hypertension and cholesterol metabolism.[13] Specifically for stroke, evidence from the literature indicates that a significant portion of risk can be attributed to common genetic factors.[13] These findings highlight both the genetic and pathological diversity underlying VaD etiology and emphasize the pivotal role of genetic predisposition in vascular brain changes.

Lewy Body dementia (DLB)

DLB displayed the lowest heritability estimate among the dementia types studied, at 34.0% (range 0-58), marking the first of its kind. These findings align with a two-stage GWAS by Guerreiro *et al.*[16] who found a heritable component of 36%. However, a more recent study by the same group reported a heritability estimate of 59.9%, based on genome-wide complex trait analysis.[17] Interestingly, our data reveal a broad range of heritability estimates, suggesting that some families may possess a rare genetic haplotype that is associated with an increased risk for DLB.[18] While genetic research on DLB is still emerging, the lower heritability estimate aligns with the current understanding that DLB has a more complex etiology involving both genetic and environmental factors.[19] This suggests that very large GWAS will be required to identify variants and candidate genes contributing to DLB risk, or alternatively, genetic studies could target rare variants within families with multiple affected members.

Ischemic (iCVA) and hemorrhagic stroke (hCVA)

We found a moderate genetic component for iCVA (27.4%, range 6-59) and hCVA (29.1%, range 8-57). These findings align with twin studies for stroke death (32%, range 4-47)[20] and subarachnoid hemorrhage (41%, range 23.7-55.5),[21] as well as genome-wide complex trait analysis for iCVA (37.9%, *SE* 5.2)[12] and hCVA (29%, *SE* 11).[22] These findings underscore the polygenic nature of stroke, aligning with existing literature.[13]

The substantial shared environmental effect we found (15.9%, and 14.9%) is supported by literature that has identified a range of environmental risk factors for stroke, such as diabetes, high cholesterol, smoking, and alcohol consumption.[23] The combination of a moderate genetic effect and a relatively high shared environmental effect on stroke highlights the need for family-based initiatives to modify this risk.

Parkinson's disease (PD)

Our findings indicate a moderate heritability estimate of 37.9% (range 6–66) for PD. This estimate is consistent with previous studies, which have reported twin heritability ranges between 28% and 44%,[24] and a SNP-based heritability of 28% (*SE* 5%).[2] The role of both rare and common genetic variants, including mutations in genes such as *SNCA*, *LRRK2*, and *PARK7*, underscore the complex interplay of genetic factors in PD etiology.[25] Additionally, while shared environmental factors were not significant in our study, environmental factors such as exposure to pesticides and smoking have been implicated to an increased risk of PD,[26,27] underscoring the multifactorial nature of the disease.

Multiple sclerosis (MS)

MS showed the lowest heritability at 10.2% (range 10-97). Twin studies indicate a heritability range between 39 and 61%,[28] and GWAS indicate a heritability of 19.2%.[29] Notably, genetic variants in genes related to immune system regulation, such as *HLA-DRB1*, have been strongly associated with MS risk.[30] To date, there is no evidence of monogenic forms of MS.[31] While these findings point to significant genetic contributions, studies have shown that MS is influenced by a combination of genetic and environmental factors.[31] Environmental factors, including viral infections, vitamin D deficiency, and smoking, also seem to play critical roles in the disease's onset and progression.[31]

Amyotrophic lateral sclerosis (ALS)

Our study estimated the heritability of ALS at 72.3% (range 10-98), suggesting a significant genetic component in disease susceptibility. Our estimate aligns with previous twin estimates for *sporadic* ALS, which range from 38 to 78%,[32] and GWAS have estimated the heritability of ALS between 17 and 25%.[33] ALS is primarily associated with mutations in genes such as *SOD1*, *TARDP*, and *FUS*.[32] Notably, a monogenic cause is identified in approximately 70% of familial ALS cases and in 10% of sporadic cases, with the most frequent genetic cause being a noncoding hexanucleotide repeat expansion in the *C9ORF72* gene.[34] While most ALS cases are classified as sporadic, genetic predisposition likely interacts with environmental factors, such as exposure to lead, pesticides, and smoking[35] – factors not directly examined in our study.

Supplementary References

1. Gatz M, Reynolds CA, Fratiglioni L, et al. Role of genes and environments for explaining Alzheimer disease. *Arch Gen Psychiatry*. 2006;63(2):168-174. doi:10.1001/ARCHPSYC.63.2.168
2. Guerreiro R, Escott-Price V, Darwent L, et al. Genome-wide analysis of genetic correlation in dementia with Lewy bodies, Parkinson's and Alzheimer's diseases. *Neurobiol Aging*. 2016;38:214.e7-214.e10. doi:10.1016/j.neurobiolaging.2015.10.028
3. Anttila V, Bulik-Sullivan B, Finucane HK, et al. Analysis of shared heritability in common disorders of the brain. *Science (1979)*. 2018;360(6395). doi:10.1126/science.aap8757
4. Tijms BM, Vromen EM, Mjaavatten O, et al. Large-scale cerebrospinal fluid proteomic analysis in Alzheimer's disease patients reveals five molecular subtypes with distinct genetic risk profiles. *Nat Aging*. 2024;4:33-47. doi:10.1101/2023.05.10.23289793
5. Cummings J, Zhou Y, Lee G, Zhong K, Fonseca J, Cheng F. Alzheimer's disease drug development pipeline: 2023. *Alzheimer's & Dementia: Translational Research & Clinical Interventions*. Published online May 25, 2023. doi:10.1002/trc2.12385
6. Greaves C V., Rohrer JD. An update on genetic frontotemporal dementia. *J Neurol*. 2019;266(8):2075. doi:10.1007/S00415-019-09363-4
7. Reus LM, Jansen IE, Mol MO, et al. Genome-wide association study of frontotemporal dementia identifies a C9ORF72 haplotype with a median of 12-G4C2 repeats that predisposes to pathological repeat expansions. *Transl Psychiatry*. 2021;11(1). doi:10.1038/s41398-021-01577-3

8. Pottier C, Ren Y, Perkerson RB, et al. Genome-wide analyses as part of the international FTLD-TDP whole genome sequencing consortium reveals novel disease risk factors and increases support for immune dysfunction in FTLD. *Acta Neuropathol*. 2019;137(6):879. doi:10.1007/S00401-019-01962-9
9. Seelaar H, Kamphorst W, Rosso SM, et al. Distinct genetic forms of frontotemporal dementia. *Neurology*. 2008;71(16):1220-1226. doi:10.1212/01.WNL.0000319702.37497.72/ASSET/81349834-7ABD-429D-90D5-CE8B750755E6/ASSETS/GRAPHIC/4FSM2.GIF
10. Mina Bergem AL, Engedal K, Kringlen E. The Role of Heredity in Late-Onset Alzheimer Disease and Vascular Dementia A Twin Study Background: This study compares the relative impor-tance of heredity and environment in the development. *Arch Gen Psychiatry*. 1997;54.
11. Carmelli D, DeCarli C, Swan GE, et al. Evidence for genetic variance in white matter hyperintensity volume in normal elderly male twins. *Stroke*. 1998;29(6):1177-1181. doi:10.1161/01.STR.29.6.1177/FORMAT/EPUB
12. Bevan S, Traylor M, Adib-Samii P, et al. Genetic heritability of ischemic stroke and the contribution of previously reported candidate gene and genomewide associations. *Stroke*. 2012;43:3161-3167.
13. The Mega Vascular Cognitive Impairment and Dementia (MEGAVCID) consortium. A genome-wide association meta-analysis of all-cause and vascular dementia. *Alzheimer's and Dementia*. 2024;20:5973-5995. doi:10.1002/alz.14115
14. Markus HS, Schmidt R. Genetics of vascular cognitive impairment. *Stroke*. 2019;50(3):765-772. doi:10.1161/STROKEAHA.118.020379
15. Pathan N, Kharod MK, Nawab S, Scipio M Di, Par G, Chong M. Genetic Determinants of Vascular Dementia. *Canadian Journal of Cardiology*. Published online 2024. doi:10.1016/j.cjca.2024.03.025
16. Guerreiro R, Ross OA, Kun-Rodrigues C, et al. Investigating the genetic architecture of dementia with Lewy bodies: a two-stage genome-wide association study. *Lancet Neurol*. 2018;17(1):64-74. doi:10.1016/S1474-4422(17)30400-3
17. Guerreiro R, Escott-Price V, Hernandez DG, et al. Heritability and genetic variance of dementia with Lewy bodies. *Neurobiol Dis*. 2019;127:492. doi:10.1016/J.NBD.2019.04.004

18. Labbé C, Heckman MG, Lorenzo-Betancor O, et al. MAPT haplotype H1G is associated with increased risk of dementia with Lewy bodies. *Alzheimers Dementia*. 2016;(12). doi:10.1016/j.jalz.2016.05.002
19. An D, Xu Y. Environmental risk factors provoke new thinking for prevention and treatment of dementia with Lewy bodies. *Heliyon*. 2024;10(9). doi:10.1016/j.heliyon.2024.e30175
20. Bak S, Gaist D, Søren HS, Skytthe A, Christensen K. Genetic Liability in Stroke: A Long-Term Follow-Up Study of Danish Twins. *Stroke*. 2002;33:769-774. doi:10.1161/hs0302.103619
21. Korja M, Silventoinen K, McCarron P, et al. Genetic epidemiology of spontaneous subarachnoid hemorrhage: Nordic Twin Study. *Stroke*. 2010;41(11):2458-2462. doi:10.1161/STROKEAHA.110.586420
22. Devan WJ, Falcone GJ, Anderson CD, et al. Heritability estimates identify a substantial genetic contribution to risk and outcome of intracerebral hemorrhage. *Stroke*. 2013;44(6):1578-1583. doi:10.1161/STROKEAHA.111.000089/-/DC1
23. Carr FJ, Mcbride MW, Carswell HVO, et al. Genetic Aspects of Stroke: Human and Experimental Studies. *Journal of Cerebral Blood Flow & Metabolism*. 2002;22(7):767-773. doi:10.1097/00004647-200207000-00001
24. Polderman TJC, Benyamin B, De Leeuw CA, et al. Meta-analysis of the heritability of human traits based on fifty years of twin studies. *Nat Genet*. 2015;47. doi:10.1038/ng.3285
25. Deng H, Wang P, Jankovic J. The genetics of Parkinson disease. *Ageing Res Rev*. 2018;42(September):72-85. doi:10.1016/j.arr.2017.12.007
26. Elbaz A, Clavel J, Rathouz PJ, et al. Professional Exposure to Pesticides and Parkinson Disease. *Ann Neurol*. 2009;66:494-504. doi:10.1002/ana.21717
27. Huang Y, Chen Q, Wang Z, et al. Risk factors associated with age at onset of Parkinson's disease in the UK Biobank. *NPJ Parkinsons Dis*. 2024;3(10). doi:10.1038/s41531-023-00623-9
28. Fagnani C, Neale MC, Nisticò L, et al. Twin studies in multiple sclerosis: A meta-estimation of heritability and environmentality. *Multiple Sclerosis*. 2015;21(11):1404-1413. doi:10.1177/1352458514564492
29. Patsopoulos NA, Baranzini SE, Santaniello A, et al. Multiple sclerosis genomic map implicates peripheral immune cells and microglia in susceptibility. *Science (1979)*. 2019;365(6460). doi:10.1126/science.aav7188

30. Hollenbach JA, Oksenberg JR. The Immunogenetics of Multiple Sclerosis: A Comprehensive Review. *J Autoimmun*. 2015;64:13-25. doi:10.1016/j.jaut.2015.06.010
31. Patsopoulos NA. Genetics of Multiple Sclerosis: An Overview and New Directions. Published online 2018. doi:10.1101/cshperspect.a028951
32. Al-Chalabi A, Fang F, Hanby MF, et al. An estimate of amyotrophic lateral sclerosis heritability using twin data. *J Neurol Neurosurg Psychiatry*. Published online 2010. doi:10.1136/jnnp.2010.207464
33. Keller MF, Ferrucci L, Singleton AB, et al. Genome-Wide Analysis of the Heritability of Amyotrophic Lateral Sclerosis. *JAMA Neurol*. 2014;71(9):1123. doi:10.1001/JAMANEUROL.2014.1184
34. Volk AE, Weishaupt JH, Peter ·, Andersen M, Ludolph AC, Kubisch · Christian. Current knowledge and recent insights into the genetic basis of amyotrophic lateral sclerosis Introduction and clinical aspects. *medizinische genetik*. Published online 2018. doi:10.1007/s11825-018-0185-3
35. Oskarsson B, Horton DK, Mitsumoto H. Potential Environmental Factors in Amyotrophic Lateral Sclerosis. *Neurol Clin*. 2015;33(4):877-888. doi:10.1016/j.ncl.2015.07.009
